# Pre-intervention child maltreatment risks, intervention engagement, and effects on child maltreatment risk within an RCT of MHealth and parenting intervention

**DOI:** 10.3389/fdgth.2023.1211651

**Published:** 2023-07-11

**Authors:** Kathleen M. Baggett, Betsy Davis, Connie Olwit, Edward G. Feil

**Affiliations:** ^1^ Mark Chaffin Center for Healthy Development, School of Public Health, Georgia State University, Atlanta, GA, United States; ^2^ Oregon Research Institute, Eugene, OR, United States

**Keywords:** digital MHealth, parenting support, child maltreatment, violence prevention, infant

## Abstract

**Introduction:**

Evidence-based mental health and parenting support services for mothers postpartum can reduce risk for child maltreatment. However, women suffering economic and cultural stressors disproportionately shoulder the burden of infant caregiving while experiencing profound barriers to accessing mental health and parenting services. This article reports on an MHealth and parenting intervention targeting maternal mood and positive parent practices within a randomized controlled trial, which provided a unique opportunity to view pre-intervention child maltreatment risk, its relationship to subsequent intervention engagement, and intervention engagement effects on pre-post child maltreatment risk reduction.

**Method:**

Principal component factor analysis was conducted to identify a modifiable pre-intervention child maltreatment risk construct within a combined MHealth and parenting intervention sample of 184 primarily Black mothers and their infants. An independent t-test was conducted to compare pre-intervention child maltreatment risk levels between mothers who went on to complete at least two-thirds of the intervention and those who did not. A GLM repeated measures analysis of variance was conducted to determine effects of intervention engagement on child maltreatment risk reduction.

**Results:**

Pre-intervention child maltreatment risk did not differentiate subsequent maternal intervention completion patterns. Mothers who completed two-thirds of the intervention, compared to those who did not, demonstrated significant reductions in pre-post child maltreatment risk.

**Discussion:**

Findings underscore the potential of MHealth parenting interventions to reduce substantial child maltreatment risk through service delivery addressing a range of positive parenting and behavioral health needs postpartum, a particularly vulnerable developmental period for maternal depression and child maltreatment risk.

## Introduction

The individual and societal costs of child maltreatment are extensive with deleterious effects across the life course and intergenerationally (1, 2). Economic burden of child maltreatment is estimated at $424 billion in lifetime costs incurred annually (3). Infancy is a particularly vulnerable development period comprising the most rapid pace of critical neurodevelopment across the life course (4). Moreover, as compared to other developmental periods, infants under 1 year of age experience the highest rate of child maltreatment with rates decreasing as child age increases (5). In the U.S. the most prevalent type of service contact to address child maltreatment occurs within child welfare systems after substantiation of child harm (6). Children from birth to age five comprise the largest share of children entering foster care (7). Because safe, nurturing caregiving is the cornerstone of infant survival (8, 9), infants who experience child maltreatment and unstable foster care placements, are at extraordinary risk for mortality, severe morbidities, and poor quality of life not only in infancy but throughout the life course (1, 2). Reducing child maltreatment during infancy is foundational for optimizing developmental health and well-being trajectories (8, 9). The most effective interventions for preventing future child maltreatment are those that begin before child maltreatment occurs and strengthen explicit parent practices that protect against it (10, 11). The critical importance of child maltreatment prevention services is underscored by research highlighting poor rates of differentiating substantiated from unsubstantiated reports (12) with more than 75% of children placed in foster care having received a prior unsubstantiated report (13).

Outside the child welfare system, voluntary early home visiting programs are one of the few approaches to providing positive parent support. In the absence of universal positive parent support programs in the U.S., community family support programs, such as Early Head Start and Healthy Families (14) serve parents experiencing severe stressors coalescing around poverty, which heighten risk for child maltreatment and challenge engagement in intervention (15). Examples include but are not limited to low social support (16), maternal depression (17), parenting stress (18), and high levels of distrust of service providers (19).

Structural and systemic racism exacerbate stressors, which undermine parents and increase risk or child maltreatment (5). Multiple structural and systemic barriers to accessing and engaging in culturally appropriate child maltreatment prevention, family support, and mental health services. These include non-standard, lack of affordable, safe, flexible childcare and early learning settings, lack of transportation, low paying jobs with non-standard, unpredictable, and inflexible work schedules and shift work (20–22). Moreover, historical experiences of harm within medical and mental health systems have yielded high levels of distrust (19). Consequently, extreme inequities have resulted in minoritized populations, who are most in need of support being least likely to access and engage in them (23, 24).

Digital interventions have potential to overcome access barriers to service receipt by providing 24-7 access to tailored intervention supports to improve maternal mental health and wellbeing (25, 26) and reduce child abuse potential (27). Digital interventions can include “in the moment” feedback that is context salient with low literacy demands (25). Prompts can be both automated and tailored to personal preferences, such as particular times of the day and in particular settings, as reminders for program engagement, skill practice, or for conducting wellbeing checks and providing concrete referrals for support (NIH R01 HD086894). Such approaches are particularly important for redressing service inequities and increasing engagement in child maltreatment prevention for mothers who suffer under economic and cultural stressors. These mothers disproportionately shoulder the burden of caregiving during infancy. Addressing child maltreatment risk for these mothers is crucial.

In a prior randomized controlled trial of a technology-adapted parenting intervention with low-income mothers of infants, several factors associated with financial strain, including low education, low social support, and maternal distress predicted child abuse potential (27). Mothers at highest risk for child maltreatment, as compared to those at lower risk, engaged in significantly less positive parenting behavior with their infants at pre-assessment. Mothers at higher risk for child maltreatment who engaged in higher vs. lower levels of intervention engagement demonstrated significantly greater gains in observed positive parenting support behaviors with their infants. Although this intervention study targeted parenting behavior, it did not target maternal depression.

Currently, there are very few controlled trial studies of digital interventions that target depression and positive parent practices among mothers, particularly for minoritized mothers of infants. One example of such a study is the recently completed Mom and Baby controlled trial (28, 29) in which a sample of primarily Black mothers were recruited in the urban core of a large southern city in the U.S. All mothers were experiencing depression and predominantly lived with high levels of economic distress and low levels of social support, including partner support (28, 29). Participants were randomized to one of two, parallel, virtual interventions targeting depression and parenting: Mom and Baby Net (MBN), a cognitive behavioral approach, or Depression and Developmental Awareness (DDAS), a person-centered approach. The cognitive behavioral approach focused on specific skills to improve mood and parent engagement in positive social-emotional support practices with their infants. The Person-centered approach focused on maternal awareness of depression and infant developmental milestones. Meta-analytic studies have shown that both cognitive behavioral and person-centered approaches are effective modalities of reducing maternal depression (30, 31). Digital aspects of both interventions were identical regarding number of sessions, session length, and delivery mechanisms (e.g., 24-7 program access; a total 15 Internet-based sessions including an orientation session to support motivation and facility with the app and 14 intervention content sessions). All intervention sessions were narrated to accommodate low literacy levels and included video-based learning presentation, activities, check in questions with automated feedback, self-created videos of mother-infant interactions, video-based structured coach support calls to review session content, support learning, establish a plan to apply session learning, and referrals to address mother-identified concerns outside the scope of the study intervention. All mothers received bi-weekly prompts to complete the PHQ-9 for safety monitoring relative to suicidal ideation, which resulted in immediate virtual well-being checks and electronic provision of crisis information and referrals. This study provided a unique opportunity to examine pre-intervention child maltreatment risks, their relationship to subsequent engagement intervention, and the effects of intervention engagement on change in child maltreatment risk within a sample of primarily Black mothers. Both treatment groups demonstrated high levels of intervention engagement as measured by session completion rates. On average, mothers in both interventions completed all depression content and majority of parenting content. Half the sample completed all 15 sessions (the median). Consequently, the virtual intervention sample was combined to address the following research questions: (1) What is the relationship between pre-intervention child maltreatment risk and subsequent level of engagement in mHealth parenting interventions and (2) What is the effect of intervention engagement on child maltreatment risk?

## Materials and methods

Prior to initiating human subject activity, all study procedures were approved by the Georgia State University IRB. Inclusion criteria were established to generate a sample of depressed mothers with infants (see Sample below). Recruitment strategies included community agency referrals, research staff outreach visits to community agencies and community events, and maternal self-referral, which comprised the largest segment of the sample (28, 29). Following informed consent, participants were randomized to one of two parallel, virtual interventions: Mom and Baby Net, a cognitive behavioral approach or Depression and Developmental Awareness, a person-centered support approach. The interventions were identical regarding number of sessions, session length, and delivery mechanisms, including 24-7 program access, a total of 15 sessions including an orientation to support motivation and facility with the mobile app and 14 intervention content sessions requiring low literacy level, with immediate automated feedback, and between session coach facilitation. For more information about the interventions, see Baggett et al. (28, 29). For this report, we focus on the combined intervention sample to address questions about pre-intervention child maltreatment risk, intervention engagement, and pre-post change in child maltreatment risks relative to intervention engagement.

### Sample

Study inclusion criteria were as follows: Mothers obtained a clinically elevated score (3+) on the Patient Health Questionnaire-2 [PHQ-2; (32)] at screening, they were at least 18 years old, spoke English, and lived in the local metropolitan area of a large southern city in the U.S. Exclusion criteria included any of the following at the time of screening: history of psychotic symptoms, residence in a homeless or domestic violence, shelter, mother or infant receiving intensive medical treatment, and not having permanent legal infant guardianship. The study sample consisted of 184 mothers and their infants. On average, infants were 6 months of age, and mothers were caring for more than 2 children in the home. Relative to maternal sample characteristics (see Table 1), 88% of mothers identified as Black, 84% had not earned a college degree, 85% had incomes less than 125% of the Federal Poverty Level, and 74% reported no significant other. Although clinically elevated risk for child abuse potential was not specified as inclusion criteria for the study, 80% of mothers were classified as at high risk for child abuse potential, and 53% of mothers demonstrated a pattern of defensive responding, indicating that obtained scores for child abuse potential are an underestimate of actual risk (see Table 2 and Measures section for additional information).

**Table 1:** Sample characteristics.

Variable (*N* = 184)	Value
Maternal age in years (SD); range	28.11 (5.81); 18.54–46.09
Child age in months, mean (SD); range	6.03 (2.84); .59–11.89
Number of children in the home mean (SD); range	2.63 (1.40); 1–6
Maternal race	
Black, % (*n*)	88.04% (162)
Multi-racial/other/unknown, % (*n*)	9.24% (17)
White, % (*n*)	2.73% (5)
Maternal ethnicity	
Latinx, % (*n*)*	2.73% (5)
Maternal education (<college degree), % (*n*)*	84.15% (154)
Maternal income, % </=125%	85.47% (153)
Federal poverty guideline, (*n*)**	
No significant other, % (*n*)	73.77% (135)
BCAP child abuse potential score***	
Mean, median, (SD), range	12.69 13 (6.32) 1–25
% above clinical cut-off (*n*)	80% (147)

* *n* = 183.

** *n* = 179.

*** BCAP Child Abuse Potential Clinical cut off =>9.

**Table 2:** Study variable distributional characteristics.

Variable (*N* = 184)	*M*(SD)	Range	Skew/SEskew
BCAP child abuse potential	13.45 (6.13)	1–25	−1.46
Subscale			
BCAP lie subscale	3.47 (1.77)	0–6	−1.57
PSI total score	80.85 (20.37)	36–135	1.26
PHQ9 total score	11.16 (6.51)	0–26	1.78
Session completion	10.98 (5.14)	0–15	−4.84

### Measures

The PHQ-2 was administered online to screen for depression with the established criteria of a score of 3 or higher defined as a positive depression screen. The PHQ-2 is an efficient and well-established measure with strong psychometric characteristics for identifying individuals with depression (32). At pre-intervention assessment, participants completed a demographic questionnaire to facilitate characterization of the sample for mother's age, ethnicity, race, educational level, income, significant relationship status, number of children in the home, and infant age in months.

Participant intrapersonal risk characteristics were assessed at pre-intervention and post-intervention. The Patient Health Question-9 (PHQ-9) was administered to assess depression severity (33). The PHQ-9 possesses strong psychometric properties for assessing depression severity; a score at or above 20 is suggestive of severe depression (34). Participants were also administered the Parenting Stress Index Short Form, which has demonstrated high internal consistency and sensitivity to intervention change with mothers of infants (35). The Brief Child Abuse Potential (BCAP) Inventory was used to assess child abuse potential and defensive responding. The BCAP demonstrates strong psychometric characteristics with sensitivity to known child abuse status and to defensive responding, a response pattern indicating social desirability in which respondents self-present in an excessively favorable light, denying even minor weaknesses (36, 37). Defensive response patterns are associated with increased risk for child abuse potential and can interfere with seeking out and engaging in therapeutic intervention (38). A session completion variable was constructed to assess intervention engagement. See Table 2 for study variable distributional characteristics.

### Analysis

Before examining the first research question, “What is the relationship between pre-intervention child maltreatment risks and subsequent level of engagement in MHealth parenting interventions?”, we sought to determine if a comprehensive and modifiable child maltreatment risk construct could be formed. We conducted a principal component factor analysis of the T1 maternal child maltreatment modifiable risk indicants of maternal depression, defensive responding, and parenting stress, with the goal of identifying the constellation of these modifiable risks most strongly related to child abuse potential. Subsequently, principal component factor analysis was performed, with communality estimates above .30 as our criterion to retain variables. The unit-weighted sum of the retained variables formed our maternal child maltreatment risk prior to intervention.

To address the first question, how these converged risks relate to mothers' subsequent intervention engagement, we first viewed the distribution of the engagement variable. Half the sample completed all 15 sessions (the mode), negatively skewing the distribution of scores. Therefore, a dichotomous variable was formed to create two meaningful session completion groups for use in subsequent analyses: mothers receiving core content including all unique depression content and majority of unique parenting focused content (i.e., 10 sessions or more) vs. mothers not receiving this level of intervention (i.e., less than 10 sessions). An independent *t*-test was performed to compare pre-intervention risk levels between these two groups.

To address the second question, determining the effect of intervention engagement (session completion level) on maternal child maltreatment risk change, a maternal risk factor after intervention was created using the same variables and procedures described above for maternal risk prior to intervention. A GLM repeated measures analysis of variance was then performed, with maternal risk as the repeated dependent measure and completion group as the independent variable of interest. A significant time ×  completion interaction would indicate differential change in child maltreatment risk based on intervention engagement.

## Results

Regarding the preliminary view of the convergence of mutable risk factors to arrive at a comprehensive maternal risk construct, correlational analyses were performed to determine how each child maltreatment risk (i.e., maternal depression, parenting stress, and defensiveness) converged with child abuse potential prior to intervention. All risks were strongly related to child abuse potential, with correlations ranging from *r*(182) = .60, *p* < .001 for maternal parenting stress to *r*(182) = .65, *p* < .001 for maternal depression. As such, all variables were entered into a principal component analysis, held to one factor, to form a merged child maltreatment risk factor. Communalities for all variables loading onto a single factor were well-above our criterion of .30, ranging from .64 to .77. Component loadings on the single factor were: .81 (depression), .81 (defensiveness), .81 (parenting stress), and .88 (child abuse potential). The component factor explained 68.07% of the variance in the component variables. All variables were unit-weighted and summed to create our maternal child maltreatment risk construct prior to intervention. The distribution for this risk construct was normally distributed (*M* = 108.52, SD = 30.00, Range = 38.00–189.00; Skew/Seskew = .16).

For the first question, given the need to dichotomize our session completion variable, a *t*-test was performed with our completion dichotomous variable serving as the independent variable and the merged maternal risk construct serving as the dependent measure. Mothers who subsequently completed less than 10 intervention sessions (*M* = 109.37, SD = 25.19) compared to mothers who subsequently completed 10 sessions or more (*M* = 108.60, SD = 30.65) did not display significantly different levels of risk prior to intervention (*t*(178) = .16, *p* = .87).

**Figure 1:**
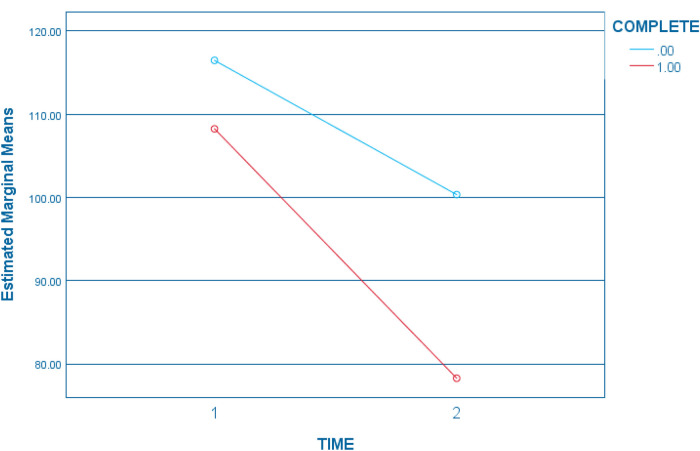
Maternal risk change by session completion level. Completion 0 = less than 10 sessions; Completion 1 = =/> 10 sessions.

For the second question, a maternal risk construct using post intervention data was created, with results indicating an equally strong convergence between maternal risks variables and child abuse potential after intervention. All risks were strongly related to child abuse potential, with correlations ranging from *r*(149) = .51, *p* < .001 for maternal parenting stress to *r*(149) = .62, *p* < .001 for maternal depression. As well, principal component factor analysis indicated strong convergence on one principal factor, with all communalities well above .30, ranging from .55 to .76. Component loadings on the single factor were: .74 (depression), .74 (defensiveness), .75 (parenting stress), and .87(child abuse potential). The single component factor explained 69.21% of the variance in the post-intervention risk variables. Unit weighting was used to create the post-invention maternal risk construct that was normally distributed (*M* = 83.71; SD = 26.40; Range = 36.00–154.00; Skew/Seskew = .41). With normally distributed maternal child maltreatment risk construct scores at pre- and post-intervention, a GLM repeated measures ANOVA was performed, with risk as the repeated dependent measure and completion group as the independent variable. Table 3 presents the within subjects contrasts for Time and Time × Completion. A significant Time × Completion group difference in risk change was observed, associated with a small effect size (*η*^2^ = .03). Mothers who completed 10 or more intervention sessions displayed a steeper reduction in risk over time than mothers who did not complete intervention sessions at this level (see Figure 1). Though no significant differences were found prior to intervention between the two completion groups, after intervention a significant difference in maternal maltreatment risk was observed. Mothers who completed 10 or more intervention sessions reported significantly lower levels of child maltreatment risk after intervention (*M* = 79.03, SD = 23.80) when compared to mothers who did not complete sessions at this level (*M* = 100.81, SD = 28.70), *t*(147) = 4.38, p < .001, equating to a large effect (*d* = .83).

**Table 3:** GLM within-subjects contrasts.

Source	Time	Type III sum of Squares	df	Mean square	*F*	Sig	Partial Eta Squared
Time	Linear	25,808.055	1	25,808.055	56.070	.000	.276
Time × complete	Linear	2,293.280	1	2,293.280	4.982	.027	.033
Error (Time)	Linear	67,660.984	147	460.279			

## Discussion

The purpose of this study was to first examine pre-intervention child maltreatment risk relative to subsequent intervention engagement, and then to understand potential engagement effects on change in child maltreatment risk within a sample of predominantly Black mothers participating a mobile MHealth and parenting intervention. Mobile intervention approaches hold promise for expanding access to crucial parenting and mental health supports by decreasing practical, psychological, and social barriers for underserved communities. They can overcome public health challenges to reaching high need populations during particularly vulnerable developmental periods when child maltreatment risk is high, parents may be more amenable to change, and intervention effects yield the most promise for harm reduction and optimization of child well-being outcomes. Moreover, such approaches are crucial for overcoming public health challenges such as the need for service greatly exceeding service provider capacity. While mobile interventions have demonstrated success in some areas, persistent challenges remain in realizing full potential for engaging those at highest need for high potential impact for themselves and their infants across the life course (39–41).

Regarding our second research question, the finding of no differences in the pre-intervention level of child maltreatment risk relative to subsequent engagement levels in intervention is surprising. High levels of child maltreatment risk experienced by mothers prior to intervention could reasonably be seen as a potential hinderance for mothers' ability to subsequently engage in intervention. But this was not found and may be due to the high level of economic stressors experienced by majority of mothers, are correlated with the modifiable risks assessed prior to intervention. We would point out, however, that the finding of no pre-intervention risk differences set a strong foundation for testing risk change over time based on intervention.

Several constraints of the present study and directions for ongoing research should be noted. First and foremost, this is a descriptive study in which participants were not randomized by high vs. low child maltreatment risk characteristics. While this is obvious, it warrants caution in interpretation of the findings. It is possible that other independent factors, not yet unexamined, could differentially effect intervention engagement. Although the absence of observed differences in intervention completion by child maltreatment risks, which are well identified within the extant literature were examined within this study, it is possible that unmeasured differences could contribute to differential completion patterns. For example, future studies could examine questions regarding potential roles of micro factor such as mobile coach behavior and fidelity.

It is encouraging that intervention feasibility, as demonstrated by high levels of parent engagement within this study, far exceeded engagement levels reported generally within home visiting interventions (42). With 80% of mothers in the sample above the clinical cut-off for classification as at high risk for child abuse potential at pre-intervention, relevance of the intervention for child welfare populations should be considered. Future studies are needed to examine MHealth parenting interventions relative to feasibility, cost, and scalability with child welfare involved families. Because severe economic distress increases risk for child welfare involvement (43), future studies are also needed to examine feasibility and scalability of MHealth parenting interventions within multi-level level approaches that include macro level interventions targeting economic sufficiency.

Another consideration is that the English-speaking inclusion criteria of the current study prevented recruitment non-English speaking participants, which constrains generalizability of these findings to marginalized groups who do not speak English. Future research should include and center such groups. Finally, beyond the focus of this manuscript, primary study outcomes are being examined within a manuscript currently under development.

## Conclusion

A recently completed randomized controlled trial provided a unique opportunity to examine: (a) pre-intervention child maltreatment risks among mothers of infants postpartum who were participating in mobile intervention targeting depression and positive parent practices, (b) pre-intervention risks relative to subsequent intervention engagement level, and (c) effects of intervention engagement on pre-post change in child maltreatment risk. Given structural and systemic racism that has severely restricted Black women's access to well-being interventions services (44) and their inclusion and engagement within controlled trials (45), the sample of predominantly Black mothers offered a rare vantage point for the above examination. Overall, our findings showed that, prior to intervention, maternal depression, parenting stress, and defensive responding were normally distributed within the sample and highly correlated with child abuse potential, producing a strong single factor yielding a merged child maltreatment construct comprised of maternal depression, parenting stress, defensive responding, and child abuse potential. Within a sample of predominantly Black mothers, experiencing high levels of economic strain and low parenting support, there were no differences in pre-intervention child maltreatment risk for mothers who went on to complete intervention sessions with all unique depression content and at least 85% of parenting content vs. those who did not. With child maltreatment risk normally distributed within sample, these findings suggest that there is perceived need for mHealth and parenting support postpartum among mothers experiencing substantial distress and low levels of trust as indicated by defensive responding, which can interfere with therapeutic engagement. Importantly, these results indicate that the use of MHealth interventions are promising strategies for provision of mental health and parenting services for mothers often not reached or engaged in services that prevent future child maltreatment before relational harm has occurred. This is of crucial importance for a demographic that has traditionally been marginalized, and, not surprisingly, experiencing elevated levels of distrust (46). Moreover, mothers who completed 10 or more intervention sessions showed significantly lower levels of child maltreatment risk after intervention when compared to mothers who did not complete sessions at this level. This finding points more broadly to the potential of MHealth parenting interventions to reduce substantial child maltreatment risk through service delivery addressing a range of positive parenting and behavioral health needs postpartum, a particularly vulnerable developmental period for maternal depression and child maltreatment risk.

## Data Availability

The raw data supporting the conclusions of this article will be made available by the authors, without undue reservation.
